# Beyond Histology: Tensiomyography as an Integrated Measure of Muscle Function in Osteoporotic and Osteoarthritic Patients

**DOI:** 10.3390/jcm15072583

**Published:** 2026-03-27

**Authors:** Chiara Greggi, Caterina Scaminaci, Manuela Montanaro, Pierpaolo Talarico, Antonio Matticari, Marco Albanese, Jure Jemec, Sergej Rozman, Alessandro Mauriello, Riccardo Iundusi, Elena Gasbarra, Umberto Tarantino

**Affiliations:** 1 Department of Orthopaedics and Traumatology, “Policlinico Tor Vergata” Foundation, Viale Oxford 81, 00133 Rome, Italy; talaricopierpaolo@gmail.com (P.T.); antonio.matticari@gmail.com (A.M.); riccardo.iundusi@uniroma2.it (R.I.); elenagasbarra@tiscali.it (E.G.); umberto.tarantino@uniroma2.it (U.T.); 2 Department of Orthopaedics and Traumatology, Policlinico Paolo Giaccone, Viale Del Vespro, 129, 90127 Palermo, Italy; caterina.scaminaci@gmail.com; 3 Department of Life Sciences, Health and Health Professions, Link Campus University, Via del Casale di S. Pio V, 44, 00165 Rome, Italy; m.montanaro@unilink.it; 4 Statistical and Actuarial Department in Italian Workers’ Compensation Authority (INAIL), Via Stefano Gradi, 55, 00144 Rome, Italy; marco.albanese@uniroma2.eu; 5 TMG-BMC d.o.o., Stihova Ulica 24, 1000 Ljubljana, Slovenia; jure.jemec@tmg.si (J.J.); sergej.rozman@tmg.si (S.R.); 6 Department of Experimental Medicine, University of Rome Tor Vergata, Via Montpellier 1, 00133 Rome, Italy; alessandro.mauriello@uniroma2.it; 7 Department of Clinical Sciences and Translational Medicine, University of Rome Tor Vergata, Via Montpellier 1, 00133 Rome, Italy; 8 Faculty of Medicine and Surgery, University “Our Lady of Good Counsel”, Rruga Dritan Hoxha, 1000 Tirana, Albania

**Keywords:** osteoporosis, osteoarthritis, sarcopenia, histology, tensiomyography, muscle function

## Abstract

**Background/Objectives:** Osteoporosis and osteoarthritis are age-related musculoskeletal disorders with a high socio-health burden, affecting both healthcare systems and individuals’ quality of life. Both conditions are generally accompanied by a concomitant decline in muscle mass and strength, referred to as sarcopenia. In this context, tensiomyography emerges as a novel, non-invasive potential diagnostic strategy for assessing muscle quality, as this parameter influences the progression of both conditions. **Methods**: Histomorphometric and immunohistochemical analyses were performed on *vastus lateralis* muscle tissue obtained from patients undergoing surgery for femoral fracture affected by osteoporosis or osteopenia, patients operated for hip osteoarthritis, and patients undergoing hip arthroplasty for osteoarthritis, concomitantly affected by osteoporosis or osteopenia. In addition, muscle function was assessed in these patients using tensiomyographic analysis. **Results**: In osteoarthritic, osteoporotic, and osteopenic patients, a reduction in muscle quality and function was observed compared with the other two experimental groups, indicating an unfavorable effect of the coexistence of the two conditions on the muscular component. Furthermore, contraction time (Tc) measured by tensiomyography was negatively correlated with lumbar spine bone mineral density values and positively correlated with the percentage of type II muscle fibers. **Conclusions**: This study highlights how tensiomyography may represent a valuable non-invasive diagnostic strategy for assessing muscle status in osteoporotic and osteoarthritic patients, as it is able to detect muscle alterations that parallel the worsening of bone status and that cannot be inferred from simple biopsy analysis. Thus, tensiomyography could be considered a practical adjunct tool in the clinical assessment of musculoskeletal frailty.

## 1. Introduction

Degenerative musculoskeletal disorders, such as osteoporosis and osteoarthritis, are increasingly emerging due to their high prevalence among elderly individuals, as well as their disabling and multidimensional impact on quality of life, autonomy, and caregiving burden [1,2]. Osteoporosis is a systemic skeletal disease characterized by a progressive reduction in bone mass and alterations in bone microarchitecture, resulting in increased bone fragility and a predisposition to fractures even from minor trauma [3]. Osteoarthritis, on the other hand, is a chronic degenerative joint disease characterized by degradation of articular cartilage, remodeling of subchondral bone, osteophyte formation, and alterations of other joint structures, leading to pain, stiffness, and functional limitation [4].

Traditionally considered distinct conditions, osteoporosis and osteoarthritis have recently garnered growing interest for their potential shared pathophysiological mechanisms, their interactions with adjacent soft tissues, and, in particular, their connection with muscle function [5]. Indeed, muscle atrophy and fiber-type changes accompanying age-related functional decline, collectively referred to as sarcopenia, appear to play a key role in the pathogenesis and clinical progression of both osteoporosis and osteoarthritis [6,7]. This condition is a progressive, generalized muscle syndrome characterized by loss of muscle mass, strength, and function, physiologically associated with aging, but potentially accelerated by pathological, nutritional, and environmental factors [8].

In this context, advanced instrumental diagnostics and histomorphometric analysis of muscle tissue emerge as highly relevant investigative tools. Non-invasive techniques, such as tensiomyography (TMG), allow precise assessment of muscle contractile properties, fiber composition, and organization. Specifically, this technique measures muscle contractile properties by detecting mechanical responses related to radial displacement of the muscle belly following a single electrical stimulus [9]. TMG has applications in sports medicine, physiotherapy, and clinical research, and may offer new opportunities for early diagnosis, risk stratification, and personalized therapeutic strategies in osteoporotic and osteoarthritic patients [10].

The aim of the present study was to investigate histological and functional differences in muscle tissue among patients with femoral fracture affected by osteoporosis or osteopenia (OP/Ope), individuals affected solely by hip osteoarthritis (OA), and patients affected simultaneously by coxarthrosis and osteoporosis or osteopenia (OA/OP/Ope). Secondly, histological data were correlated with parameters measured by TMG to explore the potential use of this technique as a novel non-invasive diagnostic tool for assessing muscle quality in this patient population.

## 2. Materials and Methods

### 2.1. Subjects

The study was approved by the Ethics Committee of Policlinico Tor Vergata (protocol number #8/23; approval date 1 March 2021). Written informed consent was obtained from all participants, and all experimental procedures were conducted in accordance with the Code of Ethics of the World Medical Association (Declaration of Helsinki). Participants were allocated into three analysis groups: 15 osteoporotic/osteopenic patients (OP/Ope) who underwent surgery for fragility fractures resulting from low-energy trauma; 15 patients with osteoarthritis (OA) who underwent surgery for hip osteoarthritis; and 15 patients undergoing surgery for osteoarthritis who were also affected by osteopenia or osteoporosis (OA/OP/Ope). Exclusion criteria included the presence of malignancies, endocrine disorders affecting bone and mineral metabolism, autoimmune diseases, and bone conditions other than primary osteoporosis. Patients receiving long-term treatment with medications known to interfere with bone metabolism, as well as those undergoing sex hormone replacement therapy and/or antifracture and/or osteoanabolic treatments, were also excluded from the study. Furthermore, since the aim of the study is to compare muscle quality and function in the three patient groups, athletes were not included.

### 2.2. Clinical and Biochemical Parameters

The densitometric diagnosis of OP was established for each subject by assessing bone mineral density (BMD) using Dual-energy X-ray absorptiometry (DXA) with a Lunar DXA device (GE Healthcare, Madison, WI, USA). Lumbar spine (L1–L4) and femoral (neck and total hip) scans were performed in accordance with the manufacturer’s recommendations [11]. BMD was expressed in grams per square centimeter (g/cm^2^) and reported as the standard deviation (SD) from the mean peak bone mass (*t*-score), with a coefficient of variation of 0.7%. Measurements were obtained from the uninjured limb. A *t*-score ≥ −1 was considered normal; values between −1 and −2.5 indicated osteopenia; and values < −2.5 were diagnostic of osteoporosis. In OA patients, BMD measurements were performed on the non-dominant side, with participants positioned supine on the examination table and the lower limbs slightly abducted. The DXA examination was carried out 1 day prior to surgery in OA patients and 1 month after surgery in OP patients. Hip radiographs were obtained to confirm fractures or to evaluate hip OA. The Kellgren–Lawrence scale was used to determine OA severity [12]. All radiographs were independently evaluated by two orthopedic surgeons. Patients with a Kellgren–Lawrence (K–L) grade ≥ 2 were classified as osteoarthritic. Serum calcium, parathyroid hormone (PTH), and 25-(OH)-vitamin D levels were measured in fasting venous blood samples. To assess muscle quality, a histomorphometric analysis of muscle tissue was performed to measure muscle fiber diameter, and an immunohistochemical analysis of fast myosin was conducted to determine the percentage of type II muscle fibers (see below). The detailed clinical characteristics of the study population are summarized in Table 1.

### 2.3. Sample Collection

Biopsies of the *vastus lateralis* muscle were obtained during hip arthroplasty procedures. The samples were fixed in 4% paraformaldehyde for 24 h and subsequently embedded in paraffin for histological analysis.

### 2.4. Histomorphometric and Immunohistochemical Analyses

Muscle biopsies were fixed in 4% paraformaldehyde for 24 h and embedded in paraffin. Subsequently, 3 µm thick sections were stained with hematoxylin and eosin (H&E) (05-06002, Bio-Optica, Milan, Italy) for histomorphometric evaluation. For each biopsy sample, ten randomly selected microscopic fields were analyzed. Images were acquired at 20× magnification using a Nikon upright microscope ECLIPSE CiS (Nikon Corporation, Tokyo, Japan) connected to a Nikon digital camera. Image analysis was carried out with NIS-Elements software (version 5.30.01; Laboratory Imaging, Prague, Czech Republic), following the manufacturer’s instructions. For the histomorphometric assessment, the diameter of 200 muscle fibers per section was measured. For immunohistochemical analysis, 3 µm thick muscle sections were incubated with a monoclonal mouse anti-Fast Myosin Skeletal Heavy Chain antibody [MY-32] (ab51263, Abcam, Cambridge, UK). Sections were washed with PBS/Tween20 (pH 7.6) (UCS Diagnostic, Rome, Italy), and immunoreactivity was visualized using a horseradish peroxidase (HRP) 3,3′-diaminobenzidine (DAB) Detection Kit (UCS Diagnostic, Rome, Italy). Immunohistochemical positivity was evaluated on digital images as described above, analyzing ten fields per section at 20× magnification.

### 2.5. Tensiomyographic Measurements

Tensiomyographic measurement was carried out using a TMG device (TMG100, TMG-BMC Ltd., Ljubljana, Slovenia), consisting of an electrical stimulator, a data acquisition module, a sensor probe, electrodes, a tripod support equipped with a manipulator, and a software interface installed on a laptop computer. Before testing, the measurement site was shaved if necessary and cleaned with alcohol to reduce skin impedance. The analysis of the *vastus lateralis* muscle was performed with the subject in the supine position, with the lower limb relaxed and the knee slightly flexed (approximately 20–30°), in order to reduce passive muscle tension. Knee flexion was standardized using a foam support to ensure consistent joint positioning across participants. After identifying the point of maximal prominence of the muscle belly, generally located at approximately the midpoint between the anterior superior iliac spine and the lateral border of the patella, the displacement sensor was positioned perpendicular to the skin surface. Two self-adhesive electrodes were placed symmetrically with respect to the sensor along the longitudinal axis of the muscle, maintaining a standardized inter-electrode distance. The inter-electrode distance was fixed at 5 cm (center-to-center), and the electrode size was 5 × 5 cm. Electrical stimulation was delivered through single, brief pulses with progressively increasing intensity until maximal muscle displacement or a stable response was achieved. Stimulus duration was set at 1 ms, starting from 20 mA and increased in 10–20 mA increments up to a maximum of 100 mA or until no further increase in Dm was observed. An adequate recovery interval was allowed between successive stimulations to avoid fatigue. A resting interval of 10–15 s was maintained between stimuli, and at least two reproducible maximal responses were recorded for analysis. Tensiomyographic parameters, including maximal radial displacement of the muscle belly (Dm), delay time (Td), contraction time (Tc), sustain time (Ts), and relaxation time (Tr), were then recorded and analyzed as indicators of the contractile and mechanical properties of the muscle [13].

### 2.6. Statistical Analysis

Data were analyzed with R (version 4.4.1) statistical software. Before using statistical test procedures, distributional assumptions were evaluated through the Pearson normality test and graphical methods (histograms and Q–Q plots), given the limited sample size; a non-parametric Kruskal–Wallis test was used to test the differences between groups, and Pearson’s correlation coefficient was considered appropriate for exploring potential correlations between the study variables. Differences were considered significant for both analyses when the *p* value was < 0.05 (** *p* < 0.01).

## 3. Results

### 3.1. Clinical Characteristics of Individuals Included in the Study

The baseline characteristics of the study population (OP/Ope: *n* = 15; OA: *n* = 15; OA/OP/Ope: *n* = 15) are reported in Table 1. The ages of the OP/Ope, OA, and OA/OP/Ope groups were 70.8, 72.4, and 73.1 years, respectively, with no statistically significant differences between any of the groups. Assessment of bone mineral density (BMD) of the lumbar spine, total femur, and femoral neck, expressed as BMD and *t*-score values, showed statistically significant differences among the patient groups: OP/Ope and OA/OP/Ope patients were characterized by lower BMD and *t*-score values at the lumbar spine, femoral neck, and total femur compared with OA patients. No significant differences were observed between OP/Ope and OA/OP/Ope. In addition, no statistically significant differences were found in circulating calcium, 25-(OH)-VitD, or PTH levels among the three groups.

### 3.2. Results of Histomorphometric and Immunohistochemical Analysis

**Figure 1:**
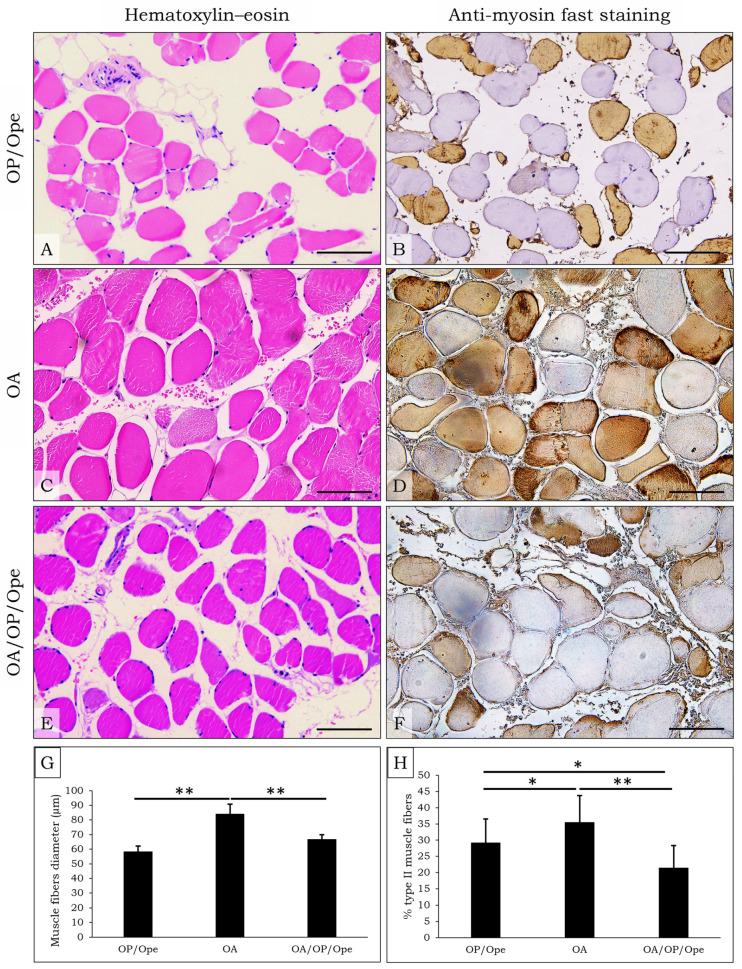
Histomorphometric and immunohistochemical analysis of muscle tissue. (**A**,**C**,**E**) Representative images at 20× magnification of Hematoxylin–Eosin-stained muscle sections from OP/Ope, OA, and OA/OP/Ope patients (scale bar = 100 µm). (**B**,**D**,**F**) Immunohistochemical staining for myosin fast (scale bar = 100 µm). H&E staining shows nuclei in blue/purple and cytoplasm in pink. Immunohistochemical investigations indicates positive signal as brown staining (DAB), highlighting the expression of the target antigen. (**G**,**H**) Graphs showing morphometric evaluation of muscle fiber diameter and percentage of type II muscle fibers in OP/Ope, OA, and OA/OP/Ope patients. Results are reported as mean ± SEM (* *p* < 0.05; ** *p* < 0.01).

Histomorphometric analysis showed that muscle fiber diameter was significantly greater in the OA experimental group compared with the OP/Ope group (*p* < 0.01) and OA/OP/Ope group (*p* < 0.01). No statistically significant difference was found between the OP/Ope and OA/OP/Ope groups. Moreover, immunohistochemical analysis of fast myosin heavy chain expression revealed that the muscle tissue of the OA/OP/Ope patient group was characterized by a statistically lower percentage of type II muscle fibers (21.5%) as compared to the OA group (35.5%, *p* < 0.01) and OP/Ope group (29.2%, *p* < 0.05). The difference in the percentage of type II fibers was also found to be significant between OA and OP/Ope groups (*p* < 0.05). Therefore, the results of both analyses t highlight how muscle tissue quality is strongly influenced by the presence of osteoporotic conditions. Indeed, qualitative morphological evaluation of H&E-stained sections suggested that the muscle tissue of OA patients, also affected by osteoporosis/osteopenia, exhibited wider inter-fiber spaces and morphological features consistent with increased adipose infiltration compared with the other two experimental groups (Figure 1).

### 3.3. Results of Tensiomyographic Measurements

The results of the tensiomyographic analysis are shown in Table 2. Since, according to the literature, there is greater agreement on the potential clinical significance of the Dm and Tc parameters, these will be the only parameters derived from the tensiomyographic analysis that will be considered in this study. Specifically, higher values of both Dm and Tc were observed in the OA/OP/Ope patient group, as compared to the other two patient groups (*p* < 0.05). Although type II fibers are typically associated with faster contraction, in pathological conditions such as OA and OP, this relationship may be altered. Indeed, this finding highlights how the concomitant presence of osteoarthritic and osteoporotic/osteopenic conditions leads to a worsening of muscle function, as increases in both parameters suggest a reduction in muscle tone and contraction velocity. For completeness of the collected data, the table also shows the other parameters; however, since there is no consensus on their potential clinical significance, they were not discussed.

### 3.4. Contraction Time (Tc) Correlates with Bone Mineral Density (BMD) of the Lumbar Spine

**Figure 2:**
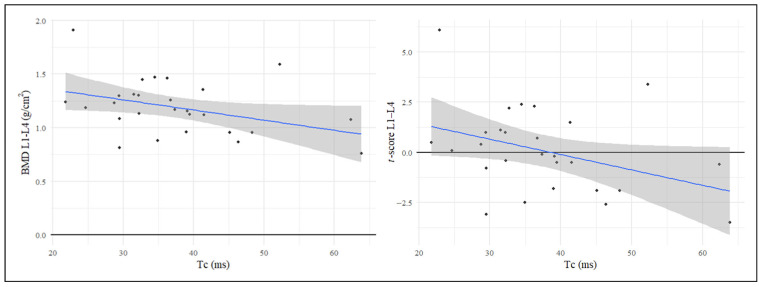
Correlation analysis between Contraction Time (Tc) and Bone Mineral Density (BMD). Pearson’s correlation coefficient was calculated. Statistical significance level set at *p* < 0.05. A negative correlation between Tc values and lumbar spine BMD (L1–L4) (r = −0.39, *p* = 0.023) was found, as well as between Tc values and *t*-score values of the L1–L4 segment (r = −0.38, *p* = 0.027).

To investigate the possible correlation between parameters indicative of bone quality status (BMD and *t*-score) and those reflecting muscle function (Dm and Tc), Pearson’s correlation coefficient was calculated and considered a statistical significance level set at *p* < 0.05. These analyses showed a negative, statistically significant correlation between Tc values and lumbar spine BMD (L1–L4) (r = −0.39, *p* = 0.023), as well as between Tc values and the *t*-score values of the L1–L4 segment (r = −0.38, *p* = 0.027). No significant correlation was observed for the Dm parameter. The correlation observed between Tc values obtained by TMG and lumbar spine BMD further supports the existence of a muscle–bone crosstalk. Moreover, these findings suggest a potential role for TMG as a tool for assessing muscle quality, as BMD appears to be directly associated with muscle contraction properties (Figure 2).

### 3.5. Contraction Time (Tc) Positively Correlates with the Percentage of Type II Fibers

**Figure 3:**
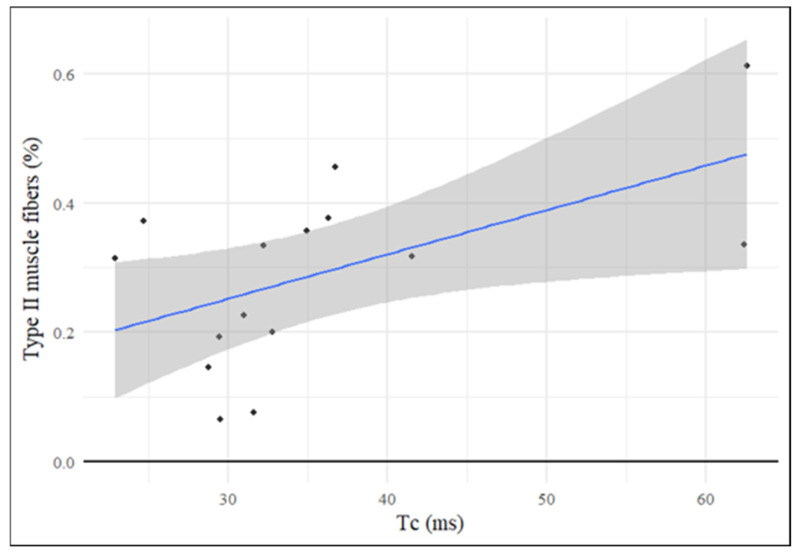
Correlation analysis between Contraction Time (Tc) and type II muscle fibers (%). Pearson’s correlation coefficient was calculated. Statistical significance level set at *p* < 0.05. A positive correlation between Tc values and percentage of type II muscle fibers (r = 0.55, *p* = 0.03) was found.

After investigating the correlation between parameters indicative of bone quality and those indicative of muscle function, the parameters obtained from TMG were correlated with the results of histomorphometric analysis of muscle tissue (fiber diameter and percentage of type II fibers) to investigate whether a linear relationship could exist between the two types of assessment (non-invasive through TMG and invasive through biopsy analysis). In this case, a statistically significant positive correlation with histomorphometric values was observed only for the Tc parameter (r = 0.55, *p* = 0.03), and not for Dm (Figure 3).

## 4. Discussion

Among the main musculoskeletal disorders associated with aging are osteoarthritis, osteoporosis, and sarcopenia. Osteoarthritis is a progressive degenerative joint disease that primarily originates in articular cartilage and, over time, affects other components of the joint. It is a highly disabling condition that causes pain and limits joint function [14]. Osteoporosis, on the other hand, is a condition characterized by a reduction in bone mass, leading to increased fragility and a higher risk of fractures [3]. Due to difficulties in walking and movement, these conditions are generally accompanied by a loss of lean mass, which translates into a decrease in muscle strength and function, collectively identified as sarcopenia [15,16]. These conditions force affected individuals into a sedentary lifestyle, leading to the development of a true state of disability and loss of independence. While clinical practice already includes rapid and validated strategies for diagnosing osteoarthritis and osteoporosis, the muscular component still receives little attention. There is currently no validated instrumental method capable of providing a clear and comprehensive assessment of muscle function in a quick and efficient manner. In this context, TMG could play a role, as it rapidly and non-invasively evaluates muscle function in terms of contractile capacity in response to an electrical stimulus [17].

The aim of the present study was therefore to evaluate, first at the histological level and then functionally, the differences in muscle quality among patients with femoral fracture affected by OP/Ope, patients affected by isolated hip OA, and patients operated for hip osteoarthritis but concurrently affected by osteoporosis or osteopenia (OA/OP/Ope).

Histomorphometric analysis of muscle tissue obtained from the *vastus lateralis* revealed that OP/Ope conditions lead to greater impairment of muscle quality, as both OP/Ope and OA/OP/Ope groups were characterized by reduced muscle fiber diameter and a lower percentage of type II fibers as compared to the OA group. Furthermore, both groups showed increased adipose tissue and greater inter-fiber space, highlighting that in these subjects, muscle quality is compromised compared to patients affected by isolated hip osteoarthritis [18]. Subsequently, a functional assessment of the same muscle was conducted using tensiomyographic analysis in terms of contractile capacity. Specifically, our focus was on Dm and Tc parameters, for which, according to prior studies in the literature, there is greater consensus regarding the reliability of their potential diagnostic significance [17,19]. Supporting the results obtained from histological evaluation, TMG analysis revealed that the muscle tissue of OA/OP/Ope patients was characterized by slower contraction velocity and lower mechanical efficiency compared to the other two experimental groups, as the values of Dm and Tc parameters were higher in these patients, who were also affected by osteoporosis/osteopenia. Several studies have reported an increase in Tc in the less active elderly population with reduced muscle function compared to young and trained subjects, reflecting an impairment of contractile properties linked to the alteration of fast fibers and the reduced neuromuscular quality typical of aging [20]. Regarding Dm, studies are more heterogeneous: this parameter can indeed be increased when referring to a specific muscle group in individuals with severely limited mobility or affected by certain pathological conditions [21].

To better characterize the potential of TMG in these patients, data obtained through this analysis were subsequently correlated with BMD and *t*-score values obtained via DXA. This investigation highlighted that Tc values were statistically significantly correlated with both BMD and *t*-score values of the lumbar spine (L1–L4). This finding suggests that TMG could represent a potential tool for assessing muscle quality, as the parameters obtained appear to reflect changes that are related to the qualitative status of bone tissue. Given the close anatomical, mechanical, and biochemical connection between bone and muscle, referred to as bone–muscle crosstalk, it is plausible that changes in bone tissue are accompanied by modifications in the functional properties of skeletal muscle, and vice versa. In this context, the observed correlation between Tc and densitometric parameters suggests that TMG may be capable of detecting such neuromuscular adaptations associated with compromised bone quality. This type of correlation was observed only for Tc, not for Dm.

To further investigate whether TMG could non-invasively assess what can otherwise be evaluated invasively through a muscle biopsy, Dm and Tc values were correlated with histomorphometric muscle data, namely fiber diameter and percentage of type II fibers. In this case as well, a statistically significant correlation was observed between Tc values and the percentage of fast fibers. Again, regarding Dm, no statistically significant correlation was found. According to the literature, Tc has been reported to correlate with the proportion of slow and fast fibers under physiological conditions, particularly in healthy, trained individuals without muscle pain or joint disorders [9,22]. In the present study, however, the coexistence of osteoarthritis and osteoporosis may alter these baseline conditions, potentially contributing to a non-linear relationship between Tc and fiber-type composition. In OA patients, joint-related inhibition and alterations in neural drive have been described and may be associated with reduced recruitment of motor units. In this context, type II fibers could be structurally preserved but functionally slower due to impaired neuromuscular activation [23,24]. Moreover, in tissues adjacent to osteoarthritic joints, including skeletal muscles involved in joint biomechanics, alterations in cellular metabolism and mitochondrial respiratory activity have been reported [25,26]. Increased extracellular matrix deposition and upregulation of profibrotic genes have also been described, potentially leading to muscle fibrosis and impaired lateral force transmission [27]. These factors might collectively influence contractile properties independently of fiber-type proportion alone. Similarly, osteoporosis has been associated with altered bone–muscle crosstalk and reduced neuromuscular quality [28]. Sarcopenia observed in this condition is generally considered to be predominantly qualitative rather than purely quantitative, as muscle functionality and fiber quality may be compromised even when fiber diameter and number are relatively preserved [29]. Therefore, in OP patients, muscle contractile behavior may not strictly reflect fiber-type distribution.

Overall, our data suggest that in patients affected by hip osteoarthritis and osteoporosis, the increase in Tc and Dm, together with a higher percentage of type II fibers, indicates a decoupling between histological composition and the mechanical behavior of the muscle. In this pathological context, therefore, the presence of fast fibers does not translate into a rapid contractile response: the relative increase in type II fiber percentage observed in these patients may reflect pathological remodeling associated with disuse. The increased percentage of type II fibers could be interpreted as a relative and compensatory phenomenon, which can coexist with reduced muscle function, suggesting that muscle quality does not necessarily follow the classic pattern of simple reduction in the percentage of fast fibers observed in the advanced stages of sarcopenia. However, while our findings demonstrate a significant association between Tc and the percentage of type II fibers, the underlying mechanisms remain unclear and warrant further investigation in studies specifically designed to explore neuromuscular and metabolic contributors to contractile alterations in osteoarthritis and osteoporosis (Table 3).

## 5. Limitations

The study has a main limitation, namely the small sample size. Larger studies are needed to refine and confirm estimates of the observed relationships. Moreover, regarding the observed non-linear relationship between Tc and type II fiber percentage, altered neural drive, mitochondrial dysfunction, extracellular matrix remodeling, and impaired bone–muscle crosstalk may contribute to this correlation, and they were not directly measured in the present study. Therefore, further mechanistic investigations specifically designed to address these pathways are needed to confirm the proposed explanation.

## 6. Conclusions

The present study highlights how TMG can detect functional alterations that are not immediately deducible from structural analysis alone. The outcome of tensiomyographic analysis represents, in fact, the integration of multiple biological levels working together. TMG simultaneously integrates neuromuscular excitability, fiber properties, the influence of connective tissue, muscle architecture, and pathological status. This type of analysis is therefore capable of detecting muscle inefficiency, which cannot be observed through histological analysis. Furthermore, TMG offers several advantages, including minimizing variability, a relatively simple measurement protocol, and the use of portable and cost-effective devices, making this diagnostic approach suitable for a wide range of clinical and research settings. Overall, this study suggests that TMG may represent a promising non-invasive tool capable of capturing functional alterations in muscle that, although related to histological characteristics, cannot be fully inferred from biopsy analysis alone. Importantly, these findings are exploratory, and their confirmation in larger, more diverse cohorts with longitudinal follow-up is critical to validate the observed associations, determine their clinical relevance, and establish TMG as a reliable tool for monitoring muscle function over time.

## Figures and Tables

**Table 1:** Clinical, histological, and biochemical characteristics of OP/Ope, OA, and OA/OP/Ope patients.

Characteristics	OP/Ope (*n* = 15)	OA (*n* = 15)	OA/OP/Ope (*n* = 15)	*p* Value
Age (years)	70.8 ± 2.5	72.4 ± 1.8	73.1 ± 3.1	ns
BMI (kg/m^2^)	26.7 ± 1.2	28.4 ± 4.8	28.1 ± 2.2	ns
DXA bone assessment				
BMD total femur (g/cm^2^)	0.8 ± 0.2	1.1 ± 0.1	0.8 ± 0.1	ns
*t*-score total femur	−1.7 ± 1.4	1.1 ± 1.0	−1.4 ± 0.9	OP/Ope vs. OA: * *p* < 0.05 OA/OP/Ope vs. OA: * *p* < 0.05 OP/Ope vs. OA/OP/Ope: ns
BMD femoral neck (g/cm^2^)	0.8 ± 0.3	1.1 ± 0.2	0.8 ± 0.1	ns
*t*-score femoral neck	−1.7 ± 2.0	0.7 ± 1.4	−2.0 ± 0.7	OP/Ope vs. OA: * *p* < 0.05 OA/OP/Ope vs. OA: * *p* < 0.05 OP/Ope vs. OA/OP/Ope: ns
BMD lumbar vertebrae L1–L4 (g/cm^2^)	0.98 ± 0.1	1.4 ± 0.2	1.1 ± 0.2	OP/Ope vs. OA: * *p* < 0.05 OA/OP/Ope vs. OA: ns OP/Ope vs. OA/OP/Ope: ns
*t*-score lumbar vertebrae L1–L4	−1.7 ± 1.2	1.6 ± 1.9	−0.9 ± 1.5	OP/Ope vs. OA: * *p* < 0.05 OA/OP/Ope vs. OA: * *p* < 0.05 OP/Ope vs. OA/OP/Ope: * *p* < 0.05
Harris Hip Score (HHS)	-	58.4 ± 13.9	48.0 ± 8.5	OA/OP/Ope vs. OA: * *p* < 0.05
Muscle histological evaluation				
Muscle fiber diameter (µm)	58.5 ± 3.7	84.1 ± 6.6	66.9 ± 2.9	OP/Ope vs. OA: ** *p* < 0.01 OA/OP/Ope vs. OA: ** *p* < 0.01 OP/Ope vs. OA/OP/Ope: ns
Type II muscle fibers (%)	29.2 ± 6.3	35.5 ± 8.2	21.5 ± 1.8	OP/Ope vs. OA: * *p* < 0.05 OA/OP/Ope vs. OA: ** *p* < 0.01 OP/Ope vs. OA/OP/Ope: * *p* < 0.05
Biochemical analyses				
Ca (mg/dL)	8.5 ± 6.2	8.7 ± 0.5	8.3 ± 0.3	ns
25-(OH)-VitD (ng/mL)	19.5 ± 9.2	20.2 ± 7.2	23.5 ± 9.1	ns
PTH (pg/mL)	68.3 ± 41.4	76.1 ± 22.4	80.4 ± 27.4	ns

BMI, body mass index; BMD, bone mineral density; PTH, parathyroid hormone; 25-(OH)-VitD, 25-hydroxyvitamin D; ns, not significant.

**Table 2:** Tensiomyographic measurements in OP/Ope, OA, and OA/OP/Ope patients.

	OP/Ope	OA	OA/OP/Ope
Dm (mm)	7.0 ± 5.1	8.9 ± 2.1	15.0 ± 2.9
Tc (ms)	32.4 ± 5.9	33.0 ± 9.1	58.8 ± 1.8
Td (ms)	30.2 ± 4.9	30.2 ± 7.3	32.1 ± 7.4
Ts (ms)	162.6 ± 9.3	158.2 ± 65.1	174.9 ± 36.0
Tr (ms)	52.9 ± 23.1	69.7 ± 33.1	76.8 ± 45.9

Dm, Maximal Displacement; Tc, Contraction Time; Td, Delay Time; Ts, Sustain Time; Tr, Relaxation Time.

**Table 3:** Schematic summary of main results.

Patients	Fibers Diameter	% Type II Muscle Fibers	TMG	Functional Implications
OA	+++	++	Tc: + Dm: +	structurally preserved but poorly functional muscle non-functional compensatory fibers hypertrophy
OP/Ope	++	+	Tc: + Dm: +	muscle quality impaired by the osteoporotic condition fast fibers present but functionally ineffective
OA/OP/Ope	++	+	Tc: +++ Dm: +++	

OA, osteoarthritis; OP, osteoporosis; Ope, osteopenia; Dm, Maximal Displacement; Tc, Contraction Time; TMG, tensiomyography. +, ++, +++ indicate grading (low, moderate, high, respectively) of the parameters dimension and presence.

## Data Availability

The original contributions presented in this study are included in the article. Further inquiries can be directed to the corresponding author(s).

## Abbreviations

The following abbreviations are used in this manuscript:

OA	Osteoarthritis
OP	Osteoporosis
Ope	Osteopenia
TMG	Tensiomyography
BMD	Bone Mineral Density
HHS	Harris Hip Score
PTH	Parathyroid Hormone
Dm	Maximum Displacement
Tc	Contraction Time
Td	Delay Time
Ts	Sustain Time
Tr	Relaxation Time
